# A Joint Gesture-Identity Recognition Framework Based on 4D Millimeter-Wave Radar Sensing

**DOI:** 10.3390/s25237249

**Published:** 2025-11-27

**Authors:** Yifan Wu, Li Wu, Taiyang Hu, Zelong Xiao, Jinyu Zhang, Mengxuan Xiao

**Affiliations:** School of Electronic and Optical Engineering, Nanjing University of Science and Technology, Nanjing 210094, China; wuyifan@njust.edu.cn (Y.W.); tyhu@njust.edu.cn (T.H.); zelongxiao@njust.edu.cn (Z.X.); zhangjinyu@njust.edu.cn (J.Z.); mengxuanxiao@njust.edu.cn (M.X.)

**Keywords:** millimeter wave radar, gesture recognition, identification, multi-task recognition, feature fusion

## Abstract

Gestures serve as an intuitive and natural medium for conveying human intent and personal identity, offering a convenient, contactless, and privacy-preserving interaction modality for human–computer interaction (HCI) systems. This paper proposes a radar-based multimodal framework for joint gesture and identity recognition, aimed at enhancing performance in radar-based gesture-identity recognition tasks. First, a robust preprocessing and multimodal feature extraction method is introduced, which integrates gesture-range-based valid frame detection with clutter suppression, enabling the extraction of multidimensional gesture features including micro-Doppler maps (MDMs), elevation–time maps (ETMs), and azimuth–time maps (ATMs). Next, a novel Joint Recognition Framework with Cross-Modal Attention Fusion (JRF-CMAF) is proposed, which incorporates Adaptive Rectification Blocks (ARBs) to dynamically leverage the complementary and correlated information across modalities. Extensive experiments were conducted on a custom radar gesture dataset collected from 7 volunteers performing 7 distinct gestures. The proposed JRF-CMAF achieves accuracies of 99.76%, 97.57%, and 96.84% in gesture recognition, identity recognition, and joint recognition tasks, respectively. Compared with conventional gesture recognition approaches and existing radar-based identity recognition methods, it attains the highest overall recognition accuracy.

## 1. Introduction

With the rapid advancement of sensor and artificial intelligence technologies [1,2,3,4], application scenarios such as smart homes [5,6,7], intelligent cockpits [8,9], and healthcare systems [10,11] are placing increasingly higher demands on HCI. Traditional interaction modalities such as touch and voice control present limitations in practical deployment. Touch-based interfaces inherently require direct physical contact, which may be unhygienic or impractical in shared or public environments. Meanwhile, voice-based interaction can be inconvenient in noisy surroundings or multi-user scenarios, where background interference, overlapping commands, and privacy concerns can significantly degrade usability [12]. At the same time, the growing demand for personalized and customized services calls for systems that not only accurately interpret user intent but also identify user identity to enable advanced functionalities such as personalized recommendations, access control, and behavioral modeling. Hand gestures, as a natural and intuitive form of communication, are capable of conveying rich control intentions [13,14]. Importantly, even when performing the same semantic gesture, individuals exhibit distinguishable dynamic patterns due to differences in hand structure, motion amplitude, and movement trajectories [15,16,17]. These patterns can be sensed, captured, and used to build identity-specific biometric models. Therefore, a joint recognition approach that integrates gesture intention recognition with identity authentication offers a highly adaptive interaction paradigm for IoT applications, enabling seamless and intelligent user experiences in complex and dynamic environments.

Early gesture data acquisition methods predominantly relied on wearable devices and vision-based sensors. Wearable devices, such as data gloves [18], smart rings [19] and wristbands [20], can achieve high recognition accuracy; however, they are constrained by complex operating conditions and inconvenient setups, often requiring extensive wiring, which hinders usability and increases cost [21]. In contrast, vision-based contactless gesture recognition systems [22,23] identify hand gestures by capturing visual features such as shape and contour using cameras. Despite their effectiveness, such systems require well-lit environments and may raise concerns regarding user privacy. To address the limitations of these approaches, gesture recognition based on millimeter-wave (mmWave) radar has emerged as a promising alternative due to its all-weather, all-day sensing capabilities, lower system cost, and inherent privacy protection advantages. Fan et al. [24] developed a Doppler radar sensor with dual receiving channels capable of time-domain remote imaging of hand and finger movements, enabling mouse-like functionalities. Liang et al. [25] proposed a continuous-wave interferometric radar approach, utilizing three sets of empirical features extracted from micro-Doppler and interferometric spectrograms for gesture classification. Frequency Modulated Continuous Wave Multiple-Input Multiple-Output (FMCW-MIMO) radar, with its advantages of wide bandwidth and high range–velocity resolution, has been extensively studied and applied in gesture recognition. Google developed the Soli gesture dataset [26] based on an FMCW-MIMO radar system, extracting range–doppler Maps (RDMs) from multiple receiving channels and employing Hidden Markov Models and Random Forest classifiers to achieve effective gesture recognition. Building on this foundation, Zhang et al. [27] and Wang [28] further introduced three-dimensional convolutional neural networks and recurrent neural networks to capture the temporal information embedded in multi-frame RDM sequences. However, these approaches have not fully exploited the angular resolution capability of MIMO radar. To address this limitation, Chmurski et al. [29] and Ahmed et al. [30] extracted multidimensional features, including angle, range, and velocity, as inputs to recognition networks, thereby improving gesture recognition performance through increased feature dimensionality. Nonetheless, most existing methods treat these feature as independent channels and simply concatenate them, without adequately leveraging the intrinsic correlations among multimodal features. Fully exploiting and fusing these inter-modal relationships forms one of the key focuses of this paper.

Although radar-based gesture recognition has been extensively studied, to the best of our knowledge, there is currently no effective solution capable of directly utilizing radar gesture data for identity recognition. Existing radar-based identity recognition studies primarily rely on specific physiological modalities. For instance, the VocalPrint [31] system utilizes millimeter-wave radar to capture skin surface reflections in the throat region during speech for identity recognition. Similarly, Y. Dong [32] proposed an identity recognition method based on vocal cord vibrations and lip movement features. The M-Auth system [33] achieves multi-user authentication by extracting distinctive breathing patterns from radar signals. Zhao [34] proposes a framework called Multimodal Neural Network Enabling User Identity Verification (MN-UIV) for enabling user identity verification using static and dynamic information via millimeter wave radar. However, these approaches depend on specific physiological activities and differ significantly from gesture recognition tasks in terms of data modality and acquisition methods, making them difficult to integrate directly into existing radar-based gesture recognition frameworks. Therefore, our work further investigates how to effectively exploit the individual-specific characteristics embedded in radar gesture signals, enabling identity recognition based solely on gesture radar data without requiring additional physiological measurements.

In summary, this paper proposes a novel radar-based gesture-identity joint recognition framework and our major contributions are summarized as follows:

- We introduce an innovative radar-based gesture echo multimodal feature extraction pipeline, which includes zero-Doppler filtering, gesture execution distance detection, and valid frame extraction. This pipeline enables the extraction of gesture MDMs, ETMs, and ATMs in cluttered environments, capturing the motion and spatial characteristics of gestures from multiple dimensions.
- We propose a novel network architecture named JRF-CMAF. The framework integrates specially designed ARBs into each modality’s feature extraction path, enabling dynamic fusion and mutual refinement of features at different depths. This enhances the network’s ability to learn inter-modal correlations and supports joint gesture and identity recognition through two task-specific output heads.
- We construct a radar gesture dataset comprising seven gesture classes performed by seven volunteer subjects. Through extensive experiments, including accuracy benchmarking, ablation studies, and open-set evaluation, we demonstrate that the proposed JRF-CMAF’s potential for real-world HCI applications.

This paper is organized as follows: In Section 2, radar echo model are established. Section 3 describes the proposed preprocessing methods of gesture data. Section 4 introduces the proposed JRF-CMAF. Section 5 gives the experiment results and analysis, followed by discussions in Section 6. Section 7 concludes this paper.

## 2. Radar Gesture Echo Modeling

This paper employs Frequency Modulated Continuous Wave (FMCW) radar to collect hand gesture echo signals. Based on the collected echoes, signal processing techniques are applied, and algorithms are designed to enable identity recognition functionality. FMCW radar operates by using a frequency-modulated continuous wave as the carrier signal, with its frequency-domain signal exhibiting periodic variations depending on the modulation scheme. During detection, the radar emits continuous frequency-modulated electromagnetic waves toward the target or obstacle. These waves are reflected upon encountering a target, forming echo signals. By accurately processing and analyzing the captured echoes, essential information about the target object can be extracted.

### 2.1. FMCW Radar Signal Modeling

**Figure 1:**
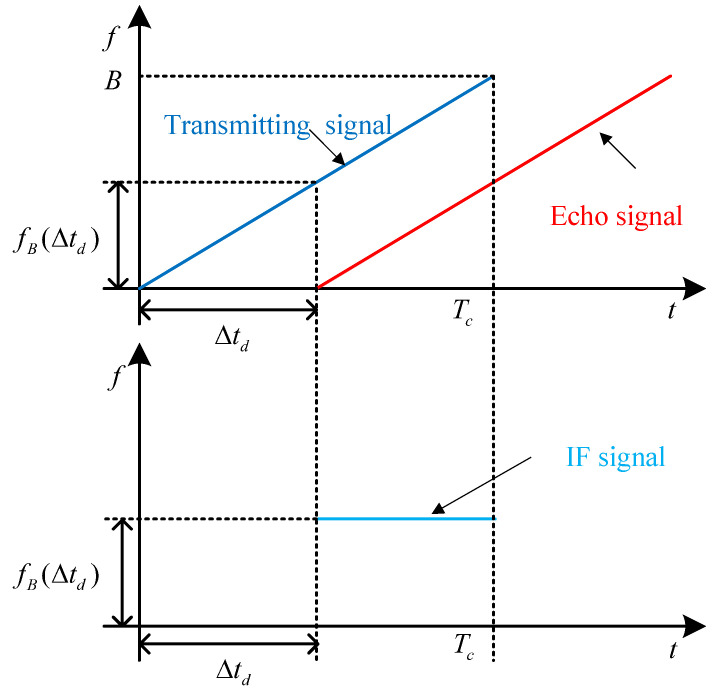
Schematic diagram of IF signal generation.

In this paper, the FMCW radar signal adopts a sawtooth wave [35] as the modulation scheme. When the radar’s transmitted signal encounters an object, it is reflected back after a certain delay, and the radar’s receiving antenna captures the echo signal. The received echo signal is then mixed with the transmitted signal, producing a constant-frequency intermediate frequency (IF) signal in their overlapping region, as illustrated in Figure 1.

The relationship between the IF signal’s frequency and time can be expressed as

(1) $$f_{B}\left(\Delta t_{d}\right)=\Delta t_{d}\cdot \frac{B}{T_{c}},$$

where $\Delta t_{d}$ represents the time delay between the transmitted signal and the echo signal, *B* denotes the bandwidth of the FMCW radar signal, and $T_{c}$ is the chirp period of the frequency-modulated continuous wave pulse.

Under the sawtooth wave modulation scheme, the transmitted signal of the FMCW radar can be expressed as

(2) $$s_{T}\left(t\right)=A_{T}\cos\left(2\pi f_{c}t+2\pi \int_{0}^{t}f_{T}\left(\tau \right)d\tau +\varphi_{0}\right),t\in \left[0,T_{c}\right],$$

where $f_{T}\left(\tau \right)=S\cdot \tau$ and *S* represents chirp slope of the signal. $A_{T}$ indicates the amplitude of the transmitted signal, $f_{c}$ denotes the carrier center frequency, and $\varphi_{0}$ represents the initial phase of the signal. When distance between the target and radar is *R*, and the target’s radial velocity relative to the radar is *v*, the frequency of the echo signal changes due to the time delay and Doppler frequency shift can be expressed as

(3) $$f_{R}\left(t\right)=S\cdot \left(t-\Delta t_{d}\right)+\Delta f_{d},$$

(4) $$\Delta t_{d}=\frac{2(R+vt)}{c},$$

(5) $$\Delta f_{d}=-\frac{2f_{c}v}{c},$$

where *c* represents speed of light. By combining the transmitted signal expression in (1) and accounting for the time delay $\Delta t_{d}$ and Doppler frequency shift $\Delta f_{d}$, the mixed signal $s_{IF}\left(t\right)$ can be expressed as

(6) $$\begin{array}{cc} s_{IF}\left(t\right) & =f_{LPF}\left\{s_{T}\left(t\right)\cdot s_{R}\left(t\right)\right\}\\ \; & =\frac{1}{2}A_{T}A_{R}\cos[2\pi \left(f_{c}\cdot \Delta t_{d}\right)\\ \; & +2\pi \left(S\cdot \Delta t_{d}-\Delta f_{d}\right)t],t\in \left[0,T_{c}\right], \end{array}$$

where $f_{LPF}$ denotes the frequency response function of a low-pass filter, and $A_{T}$ and $A_{R}$ represent the amplitude of transmitted signal and echo signal respectively. It can be observed that the frequency of the IF signal is influenced by the time delay $\Delta t_{d}$ and the Doppler frequency shift $\Delta f_{d}$.

### 2.2. TDM-MIMO Radar Signal Modeling

To extract the angular information associated with gesture movements, this paper employs a Time Division Multiplexing Multiple-Input Multiple-Output (TDM-MIMO) radar system consisting of 4 transmit antennas and 4 receive antennas.

**Figure 2:**
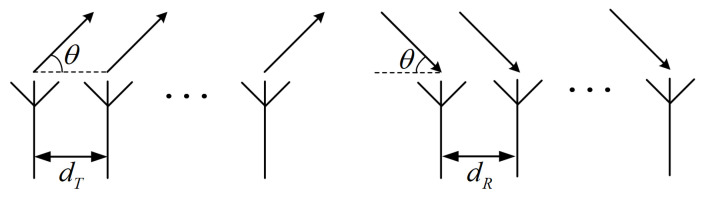
The schematic diagram of TDM-MIMO antenna array.

Based on a TDM-MIMO radar with a uniform linear array consisting of *M* transmit antennas and *N* receive antennas as shown in Figure 2, we derive the echo signal model. Considering the first transmit antenna and the first receive antenna as the reference antennas in their respective arrays, the transmitter and receiver steering vectors associated with the target’s azimuth angle θ can be expressed as

(7) $$\begin{array}{c} a\left(\theta \right)=\left[\begin{array}{cc} \; & 1\\ \; & e^{j2\pi d_{T}sin\left(\theta \right)/\lambda }\\ \; & \ldots \\ \; & e^{j2\pi \left(M-1\right)d_{T}sin\left(\theta \right)/\lambda } \end{array}\right]\\ b\left(\theta \right)=\left[\begin{array}{cc} \; & 1\\ \; & e^{j2\pi d_{R}sin\left(\theta \right)/\lambda }\\ \; & \ldots \\ \; & e^{j2\pi \left(N-1\right)d_{R}sin\left(\theta \right)/\lambda } \end{array}\right] \end{array},$$

where a(θ) represents the transmit antenna array steering vector, and b(θ) represents the receive antenna array steering vector. The variable $d_{T}$ denotes the spacing between transmit antennas, while $d_{R}$ denotes the spacing between receive antennas. Based on this configuration, the virtual array steering vector associated with the MIMO antenna arrangement can be expressed as

(8) $$\begin{array}{c} y(\theta )=a(\theta )⊗b(\theta ), \end{array}$$

where ⊗ represents the Kronecker product. Thus, for a given configuration with *M* transmit antennas and *N* receive antennas, a virtual array with M×N elements can be derived. Each element in this virtual array is represented by y(θ,n,m) within the matrix.

For the reconstructed virtual array, we further analyze the target echo signals received by each array element. The radar system transmits multiple Chirp signals continuously to obtain target range and velocity information, with these signals forming a single data frame. Assuming a target has an initial range *R* relative to the radar and a relative velocity *v*, the time delay τ can be expressed as

(9) $$\begin{array}{c} \tau \left(l\right)=\frac{2\left(R+v\left(l-1\right)T_{c}\right)}{c},l=1,\ldots ,L_{c}, \end{array}$$

where τ(l) represents the time delay corresponding to the *l*-th Chirp signal, and $L_{c}$ denotes the total number of Chirp signals in one data frame. The echo signal corresponding to the *l*-th Chirp can be expressed as

(10) $$\begin{array}{cc} s(t,l)= & A\exp(j2\pi (\left(\frac{2SR}{c}+\frac{2\nu \left(l-1\right)T_{c|}}{c}\right)t\\ \; & +f_{c}\frac{2R}{c}+\frac{2f_{c}\nu \left(l-1\right)T_{c}}{c})). \end{array}$$

Due to the spatial positions of the receive antennas, the echo signals corresponding to the same target exhibit different phases across different receive channels. The phase difference between channels is related to the target’s angle. Thus, the target echo signal received by any arbitrary virtual array element can be expressed as

(11) $$\begin{array}{c} Y(t,l,n,m,\theta )=s(t,l)\cdot y(\theta ,n,m). \end{array}$$

## 3. Preprocessing of Radar-Based Gesture Data

### 3.1. Clutter Suppression and Effective Frame Detection

Since the signal acquisition is conducted indoors, a large amount of interference echoes is present during the process. In the application of using gesture information for identity recognition, the radar’s wide radiation range may capture various non-target gesture echoes. These clutter signals may result from static environmental factors, such as the radar itself, walls, and tables, or from dynamic clutter caused by non-target objects moving within the radar beam, such as the motion of a human torso. These clutter signals are received alongside the gesture signals by the radar, potentially affecting subsequent gesture feature extraction and the final identity recognition results.

**Figure 3:**
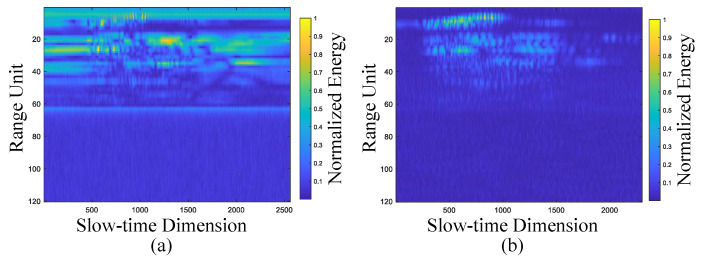
Comparison of filtering effects of RTM after four-pulse canceller. (**a**) Raw image. (**b**) Filtered image.

For each received radar echo frame, FFT is applied along the fast-time dimension to obtain a range-time map (RTM), where the color intensity reflects the magnitude of the echo energy. As illustrated in Figure 3a, the RTM of a waving-left gesture prior to clutter suppression contains significant interference, static clutter mixed with the gesture echo, making it difficult to extract meaningful gesture information. This highlights the necessity of removing stationary clutter. To remove stationary clutter, we employ a four-pulse Moving Target Indication (MTI) canceller to suppress clutter in the RTM. Compared with the traditional two-pulse canceller, the four-pulse canceller provides a deeper notch and a flatter passband response. It significantly improves the suppression of returns near zero-Doppler, making it more effective not only at filtering out stationary targets but also at attenuating low-velocity clutter. Additionally, the improved in-band uniformity enhances overall system stability. As shown in Figure 3b, after static clutter removal using the four-pulse canceller, the signal-to-clutter ratio (SCR) of the RTM is significantly improved, which facilitates the subsequent extraction of gesture-related features.

**Figure 4:**
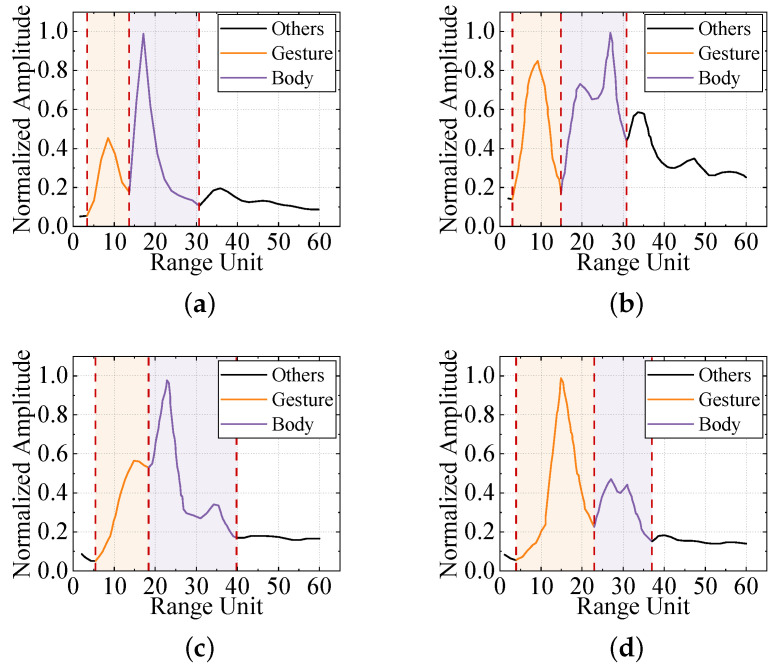
Range energy relationship diagram of different gestures performed by different subjects. (**a**) Subject 1 performs the waving left gesture. (**b**) Subject 2 performs the waving right gesture. (**c**) Subject 3 performs the waving down gesture. (**d**) Subject 4 performs the waving up gesture.

**Figure 5:**
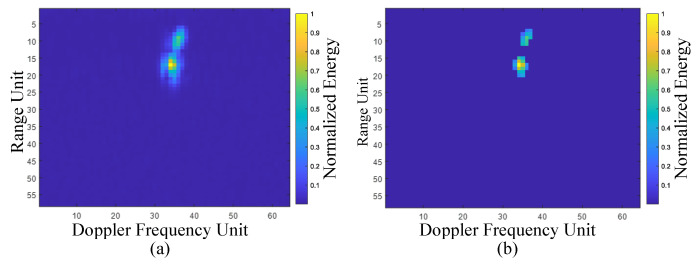
Comparison between the raw RDM and the RDM after applying the 2D-CFAR. (**a**) Raw RDM. (**b**) RDM after 2D-CFAR.

After clutter suppression, we perform incoherent integration along the fast-time axis of the filtered RTM and normalize the result. This procedure reveals the cumulative echo energy, within a single acquisition period, produced by gesture motion and by movements of the participant’s torso at each range bin. Figure 4 presents the incoherently integrated range-domain profiles for various gestures executed by different subjects. Two prominent energy peaks are evident in every profile, allowing us to segment the energy distribution into two principal regions. Considering that, during data collection, participants stood facing the radar at a distance of 0.8 m to 1.5 m and executed hand gestures within 0.2 m to 0.8 m from the radar aperture, we infer that these two segments correspond to the gesture execution range and the body-occupied range, respectively. To concentrate on gesture-induced echoes, we apply a Fourier transform along the slow-time dimension of the preprocessed RTM to obtain the range-Doppler map (RDM). Each RDM frame is then subjected to a two dimensional constant false-alarm rate (2D-CFAR) detector to isolate gesture and body signatures. Figure 5 contrasts the original and processed RDMs. Residual clutter remaining after MTI processing is effectively removed, and the energy associated with hand motion is clearly separated from that of the torso. From the Doppler perspective, micro-motions of the torso generate Doppler shifts concentrated around zero Doppler, whereas gesture movements produce Doppler components on the positive velocity side of zero Doppler, reflecting the forward motion of the hand relative to the radar.

After performing target detection on each frame of RDMs, the retained target information is weighted according to Doppler frequency and then accumulated across frames. A peak search is applied to the resulting energy curve, and the peak energy is used as a parameter to define an energy threshold. Range bins whose energy exceeds this threshold are considered part of the gesture execution range, while bins falling below the threshold are regarded as representing body motion or clutter outside the gesture range. These non-gesture bins are zeroed out in the RDM to suppress irrelevant signals. Subsequently, for each frame, the energy within the identified gesture execution range is integrated across the RDM and taken as the gesture energy feature for that frame. The resulting data is then normalized, and a threshold-based filtering step is applied to extract valid frames, ensuring that only meaningful gesture-related data is retained for further analysis.

### 3.2. Feature Extraction of Gesture Data

#### 3.2.1. Hand Gesture Micro-Doppler Feature Extraction

Micro-Doppler features generated by gesture movements are widely used in gesture recognition tasks. However, they also capture individual-specific motion characteristics, such as execution habits and dynamics, making them valuable inputs for joint gesture and identity recognition.

**Figure 6:**
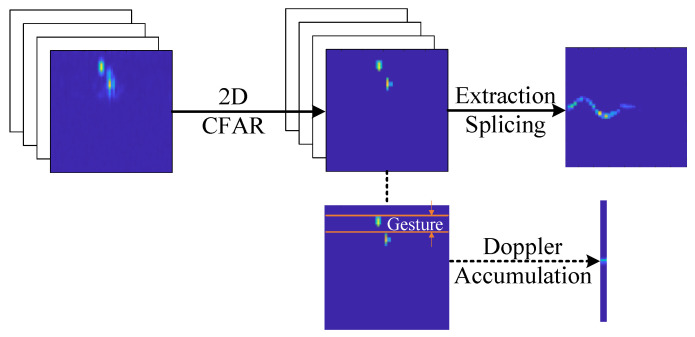
Gesture micro Doppler feature extraction process.

In the previous section, clutter suppression and gesture range detection were applied to eliminate energy components unrelated to the gesture motion, such as static background and body induced echoes. As a result, only the energy corresponding to actual hand gesture movements was preserved in the RDM. As illustrated in Figure 6, the gesture related echo energy from all valid frames is temporally concatenated to construct the MDM, which serves as a key input for subsequent recognition tasks.

#### 3.2.2. Construction of Spatiotemporal Relationship Feature Graph Based on Capon Algorithm

**Figure 7:**
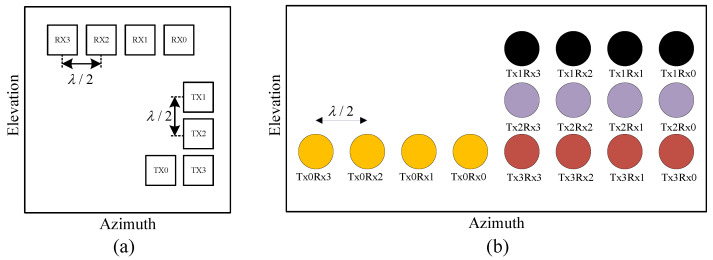
Schematic diagram of the physical and virtual antenna array arrangement in TDM-MIMO radar system. (**a**) Physical antenna array arrangement. (**b**) Virtual antenna array arrangement.

To extract the angular information embedded in hand gesture movements for subsequent recognition network input, this study employs a TDM MIMO radar system with four transmit antennas and four receive antennas. The antenna configuration is illustrated in Figure 7a, while the corresponding virtual array element distribution, derived from MIMO radar virtual array principles, is shown in Figure 7b. Based on the previous analysis of MIMO radar echo signals, it is evident that (11) inherently contains the target’s angular information. The Capon algorithm [36] is employed in this study to estimate the spatial spectrum along both the elevation and azimuth dimensions, enabling high-resolution angle-of-arrival estimation for gesture-related radar echoes.

**Figure 8:**
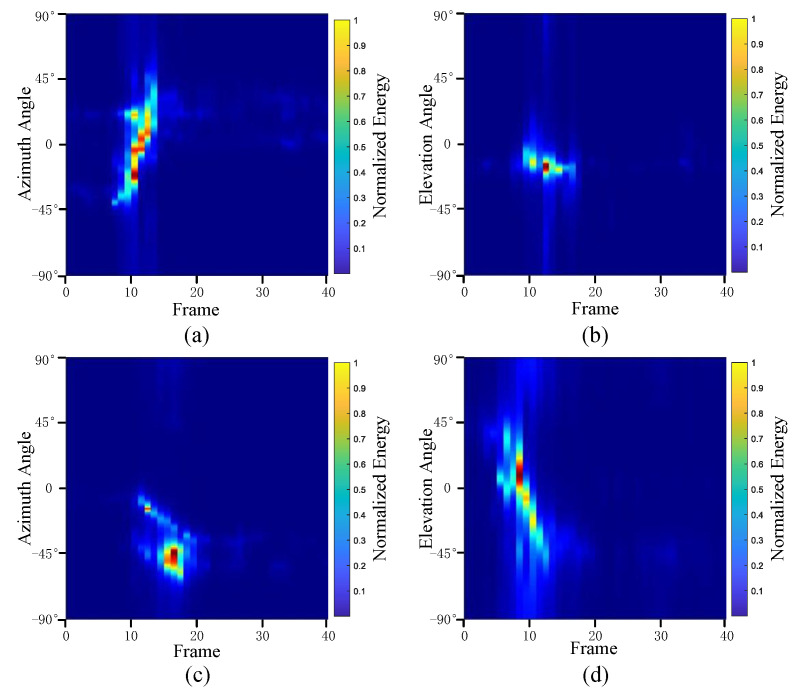
ATMs and ETMs for different gesture. (**a**) ATM with waving-right gesture. (**b**) ETM with waving-right gesture. (**c**) ATM with waving-up gesture. (**d**) ETM with waving-up gesture.

Subsequently, the non-coherent accumulation of data within the effective gesture range is performed to extract angular information for each frame. These accumulated angular features are then concatenated across frames to form the ETM and ATM. As illustrated in Figure 8, the ETM and ATM for waving-right and waving-up gestures are presented. Observing the plots, the waving-right gesture exhibits a significant change in horizontal angle over a short duration, while the elevation angle remains relatively stable, indicating a movement from legt to right relative to the radar. Conversely, the waving-up gesture shows a considerable change in elevation angle within a short period, while the horizontal angle remains stable, corresponding to a bottom-to-top movement relative to the radar.

## 4. Multi-Task Recognition Network

**Figure 9:**
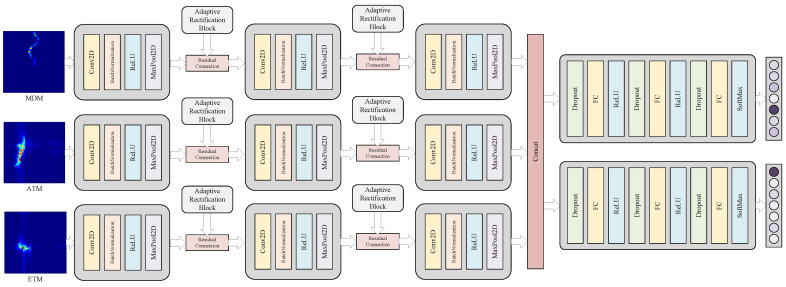
Proposed joint recognition network architecture for gesture and identity.

**Figure 10:**
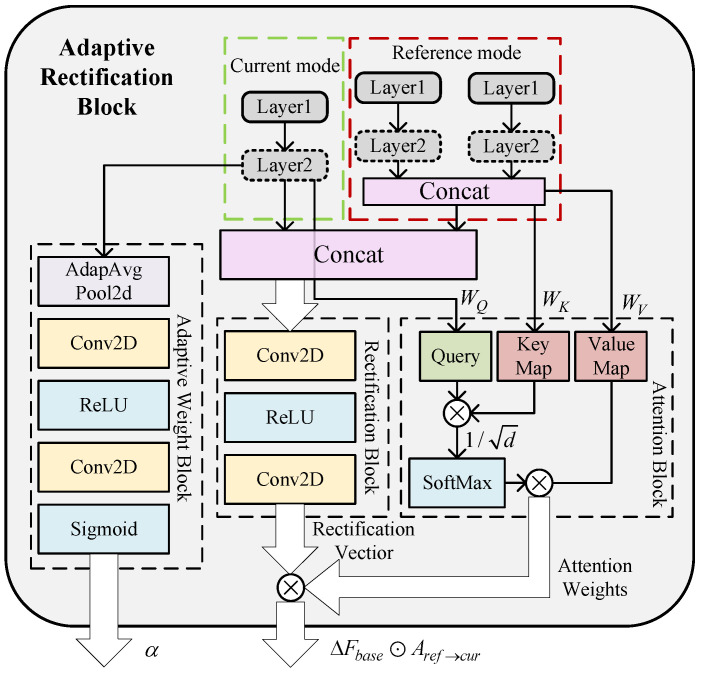
Architecture of adaptive rectification block.

To effectively integrate the multimodal information extracted from radar gesture echo signals, this paper proposes a novel multi-task recognition network architecture called JRF-CMAF. The primary objective of this architecture is to comprehensively exploit the correlations among modality-specific feature maps derived from radar gesture data, thereby enhancing the performance of both gesture and identity recognition tasks. The detailed architecture of the network is illustrated in Figure 9 and Figure 10, which indicate how features from other input modalities are integrated and refined during the convolutional processing of the current modality.

The proposed JRF-CMAF architecture consists of two main components, namely a shared feature extraction module for processing multimodal inputs and task-specific recognition output modules. The shared feature extraction modules are designed to learn and capture generalized representations of gesture-related features across modalities. In contrast, the task-specific recognition output modules address variations unique to each recognition task and enhance the network’s ability to generalize across different objectives, such as gesture classification and identity recognition.

In the shared feature extraction module for processing multimodal inputs, each modality is represented as a two-dimensional image. Therefore, we adopt a multi-layer convolutional neural network (CNN) as the foundational framework. Each input modality is passed through its corresponding shared convolutional layers, where multiple convolution kernels of varying sizes are employed to effectively capture multi-scale and hierarchical features. This design enhances the network’s capacity to understand and represent image-based data, thereby providing more comprehensive and accurate feature representations for the recognition tasks. To mitigate distributional inconsistencies across hidden layers, batch normalization is applied after each convolutional layer, improving both the sensitivity and efficiency of the network. The extracted features are then activated using the ReLU function, followed by max pooling to abstract salient gesture features. This process expands the receptive field, reinforces translation invariance, and reduces the complexity of network optimization.

Since the three input modalities of the network are not mutually independent but instead exhibit strong interdependence, it is crucial to model their cross-modal relationships. To address the discrepancies in spatial-temporal resolution and semantic granularity across modalities, this paper introduces a novel modality fusion and correction block integrated into the feature extraction process called adaptive rectification block (ARB). Specifically, for each target modality, the remaining modalities are treated as reference modalities. The corresponding feature maps from these reference modalities at the same hierarchical level are processed through a lightweight convolutional network to generate a modality-specific correction vector, which is then used to refine the features of the target modality. The architecture of ARB is shown in Figure 10 and the formulation of this block is given as

(12) $$\Delta F_{base}=Conv\left(\left[F_{cur};F_{ref}\right]\right),$$

where $F_{\mathrm{cur}}$ and $F_{\mathrm{ref}}$ represent the feature maps of the modality to be corrected and the reference modality, respectively, at a given convolutional layer. The operator Conv(·) denotes the transformation function implemented by the lightweight convolutional network used to generate the base correction matrix.

To further align the local semantic representations between the target and reference modalities, a cross-modal attention mechanism is employed. This mechanism dynamically aligns local dependencies by treating the current feature map $F_{\mathrm{cur}}$ as the query set *Q*, while using the feature maps from the reference modalities to generate the corresponding key *K* and value *V* sets. The similarity matrix between *Q* and *K* is then computed and passed through a scaling and normalization step to yield the attention weight matrix $A_{ref\to cur}$. The attention calculation is formulated as

(13) $$\begin{array}{ccc} Q=F_{cur}\cdot W_{Q} & K=F_{ref}\cdot W_{K} & V=F_{ref}\cdot W_{V}, \end{array}$$

(14) $$A_{ref\to cur}=Soft\max\left(\frac{QK^{T}}{\sqrt{d}}\right)\cdot V,$$

where $W_{Q}$, $W_{K}$, and $W_{V}$ are learnable projection matrices that transform the input feature map into the query *Q*, key *K*, and value *V* representations, respectively. The parameter *d* denotes the embedding dimension of the input features. Assuming the input feature map $F_{\mathrm{cur}}\in R^{n\times d}$, the projection matrices satisfy $W_{q},W_{k},W_{v}\in R^{d\times d}$.

Additionally, a neural network with a sigmoid activation function at its output layer is employed to dynamically generate correction weights α for each reference modality. These weights are constrained within the range of 0 to 1 and determine the degree of influence each reference modality exerts during the correction process. To ensure stable gradient propagation, particularly when the cross-modal correction magnitude is small, and to enable effective training of deeper correction modules, a residual fusion strategy is adopted. Finally, the multimodal fusion and correction at the current feature extraction layer is achieved through a residual connection that integrates the dynamically generated correction term ΔF, the attention matrix *A*, and the correction weight α. This process can be formulated as

(15) $$F_{cur}^{\prime }=F_{cur}+\alpha \cdot \Delta F_{base}⊙A_{ref\to cur}.\;$$

As illustrated in Figure 9, the ARBs are applied after Layer1 and Layer2 within each modality-specific branch. This design effectively enables mutual integration and refinement of multimodal radar gesture echo information, such as micro-Doppler frequency, elevation angles, and azimuth angles, across different feature depths. Following the third convolution-pooling block, the three modality-specific feature maps, each refined through cross-modal correction, are concatenated and fed into the subsequent task-specific recognition output modules.

The task-specific recognition module is responsible for producing both gesture and identity predictions from the fused multimodal feature representation generated by the shared extraction backbone. Although both tasks operate on the same integrated feature map, their discriminative requirements differ substantially—gesture recognition primarily relies on global temporal–dynamic patterns of motion, whereas identity recognition depends more on fine-grained, subject-specific micro-motion characteristics. To accommodate these differences, two structurally identical yet independently parameterized classification heads are constructed, as depicted in Figure 9. Each classification head comprises three fully connected layers that progressively project the high-dimensional fused features into compact task-dependent semantic embeddings, while simultaneously enabling the construction of nonlinear decision boundaries suited to the respective recognition objective. To enhance robustness under the limited-sample conditions inherent in radar-based gesture–identity datasets, a Dropout layer is inserted prior to each fully connected layer, effectively mitigating overfitting and improving generalization. For gesture recognition, the final fully connected layer outputs a Softmax distribution over the seven predefined gesture categories. For identity recognition, an analogous output layer produces a Softmax distribution over the seven enrolled subjects.

It is worth noting that the final layer of the recognition output module consists of two independent fully connected layers followed by activation functions, each corresponding to one of the classification targets, gesture and identity. As a result, the learning process yields two separate loss functions, $L_{gesture}$ and $L_{identity}$, associated with the respective tasks. A straightforward approach is to combine these losses using a fixed, manually chosen weighting scheme. However, model performance can be highly sensitive to the choice of these weights, potentially resulting in a costly and inefficient hyperparameter tuning process. To address this issue, this paper adopts the uncertainty-based loss weighting method proposed by Kendall et al. [37] during training. This method formulates the total loss $L_{total}$ for the joint gesture and identity recognition task as

(16) $$L_{total}=\frac{1}{2{\sigma_{1}}^{2}}L_{gesture}+\frac{1}{2{\sigma_{2}}^{2}}L_{identity}+\log\sigma_{1}+\log\sigma_{2},\;$$

where $\sigma_{1}$ and $\sigma_{2}$ are both learnable parameters and they represent the task-dependent uncertainties for gesture recognition and identity recognition, respectively. During training, the model dynamically and automatically adjusts these parameters to balance the contributions of the two task-specific losses. This approach helps to stabilize the training process and ensures that neither task dominates the optimization, promoting more balanced and robust multi-task learning.

## 5. Experiment and Result Analysis

This section provides a detailed description of the radar gesture dataset constructed to evaluate the performance of the proposed JRF-CMAF framework in practical gesture-identity joint recognition tasks. The experimental setup, training procedures, and recognition results for both gesture classification and identity recognition are also presented based on this dataset.

### 5.1. Radar Experimental Platform and Data Acquisition

This paper utilized a radar sensor development platform which integrates the CAL60S244-IBM and Rhine AiP radar sensors. The experimental setup and parameter configurations are detailed in Table 1.

**Figure 11:**
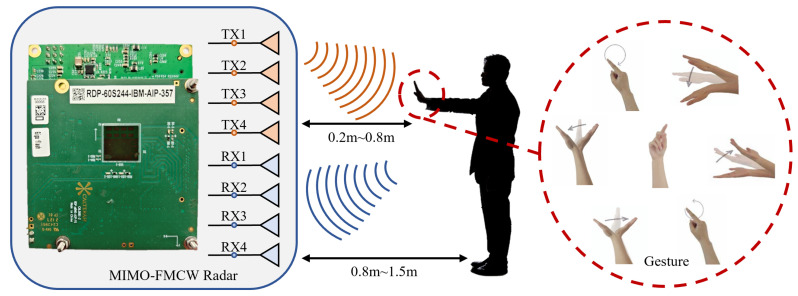
Joint gesture and identity recognition experiment and data acquisition scene based on millimeter wave radar.

To construct the experimental dataset, we recruited seven volunteers, including four males and three females. Each participant performed seven distinct gestures, with each gesture being repeated 40 times to capture a more comprehensive and diverse set of radar echo signals. Figure 11 shows the process of measuring gesture echo in this paper. Table 2 shows the specific gesture types included waving-left, waving-right, waving-down, waving-up, rotating-clockwise, rotating-counterclockwise, and finger snapping. In total, the dataset comprised 1960 recorded samples. During the collection of radar echo signals, participants were required to face the radar directly, maintaining a body-to-radar distance of 0.8 m to 1.5 m. The gesture execution range was set between 0.2 m and 0.8 m directly in front of the radar. Participants were instructed to perform gestures naturally according to their own habits while maintaining a consistent execution frequency for the same gesture. Additionally, they were asked to keep their bodies relaxed and minimize excessive movement to prevent signal interference. To enhance the reliability and diversity of the dataset, the first half and the last half sets of gesture echo data were collected on different days.

### 5.2. Result and Analysis of Joint Recognition of Gesture and Identity

According to the radar gesture echo feature extraction method described previously, each sample input to the recognition network consists of three preprocessed two-dimensional images: the MDM, ATM, and ETM representations. In these images, each pixel value corresponds to the echo signal intensity. However, due to differences in spatial resolution across modalities, the input images from different modalities vary in size. To address this, we apply bilinear interpolation to normalize all modality-specific images to a unified resolution of 64 × 64 pixels. This standardized resolution is used as the input size for the subsequent feature extraction networks for each modality.

To achieve optimal recognition performance and improve training efficiency, we conducted extensive experiments and parameter tuning. Based on these trials, we selected the network hyperparameters listed in Table 3. All subsequent experiments in this study are carried out using these hyperparameter settings as the default configuration.

To validate the effectiveness of the proposed JRF-CMAF framework, several recently introduced multi-task recognition approaches were selected for comparison [38,39], including methods specifically designed for radar echo-based multi-task recognition [34,40]. These methods were trained and evaluated on our constructed radar gesture echo dataset to compare their recognition accuracy against that of the proposed framework. All models are trained on a workstation with an Intel I9-13900k CPU and an NVIDIA Geforce 4090 GPU. All the experiments are implemented with the PyTorch 2.0.0 framework.

**Figure 12:**
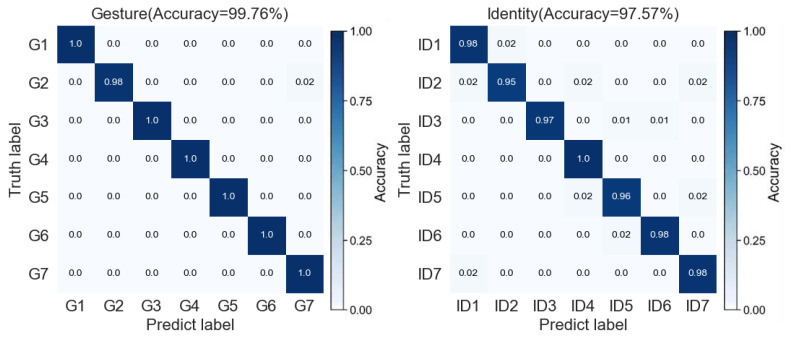
Confusion matrix of JRF-CMAF in gesture recognition and identity recognition tasks.

As shown in Table 4, the proposed JRF-CMAF consistently achieved the highest recognition accuracy across all evaluated tasks—individual gesture recognition, individual identity recognition, and joint gesture-identity recognition—when compared with all benchmark algorithms, including MN-UIV, Joint Motion classification and person Identification Convolutional Neural Network (JMI-CNN), Multilinear Relationship Networks (MRN), Convolutional Block Attention Module Convolutional Neural Network (CBAM-CNN), and the ablated version of our model without ARBs. Specifically, for the standalone gesture recognition task, JRF-CMAF outperformed the best-performing baseline by approximately 1%. In the standalone identity recognition task, it achieved an improvement of around 4%. Most notably, in the joint recognition task, the proposed method demonstrated a substantial accuracy gain of approximately 7%. These findings indicate that the proposed method delivers a significant performance advantage in the more complex joint recognition setting. Analyzing the results of the baseline methods reveals that many existing approaches tend to perform well on gesture recognition task but fail to maintain comparable accuracy when extended to the other task or the joint task. This may be due to their architectural design being inherently oriented toward gesture recognition, without the capacity to effectively extract deep individual motion patterns and identity-specific features from multimodal radar inputs. In contrast, the proposed JRF-CMAF is specifically designed to address the challenges of radar-based gesture-identity joint recognition. As illustrated in Figure 12, the confusion matrices illustrate the performance of the proposed JRF-CMAF network on both gesture and identity recognition tasks. The overall gesture recognition accuracy reaches 99.76%, with only minor misclassification observed in the G2 gesture category. For the identity recognition task, the network achieves an overall accuracy of 97.57%. Except for a few instances of confusion among certain subjects, most identity classes maintain high classification confidence. This confusion is primarily attributed to the similarity in motion patterns exhibited by different individuals when performing specific gestures, which can lead to partial feature overlap in the multimodal feature space. Nevertheless, the network effectively distinguishes individual identities by leveraging its cross-modal fusion and adaptive rectification mechanisms, which enhance its ability to extract discriminative identity-specific features from multimodal radar data.

**Figure 13:**
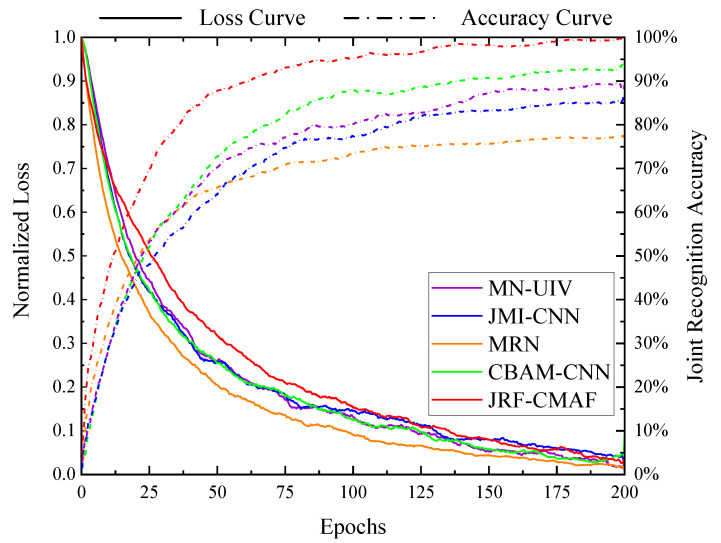
Comparison of the loss decline accuracy degree in the training process of each model.

To further evaluate the performance of the proposed framework, we present the training loss curve and validation accuracy curve over the course of training as Figure 13. During the early to mid stages of training, approximately 20 to 100 epochs, our network exhibits a relatively slower rate of loss reduction compared to other methods. We attribute this to the deeper feature learning mechanisms embedded in JRF-CMAF, which require more training iterations to construct effective cross-modal representations. Notably, despite the slower convergence of the loss during this phase, the validation accuracy of our model consistently surpasses that of the baseline. This indicates that the network is learning more discriminative and task-relevant feature representations even before the loss begins to decline rapidly. After around 100 epochs, once the feature space has been sufficiently restructured, the loss begins to decrease more rapidly and eventually converges to a level comparable to that of the comparison models. Upon convergence, the JRF-CMAF achieves a training accuracy that surpasses the optimal baseline by 5%. These training dynamics demonstrate that the proposed framework possesses a stronger feature learning capacity and ultimately converges to a higher-quality solution. However, the cross-modal correction and fusion mechanisms require adequate training duration to be fully activated and effectively utilized.

## 6. Discussion

### 6.1. Contribution of Radar Feature Modalities

To evaluate the contribution of multimodal radar inputs to the performance of joint gesture and identity recognition, we conducted a controlled modality configuration experiment. In this experiment, we systematically designed a series of input conditions by selectively enabling different combinations of radar modalities. This design allowed us to assess how each individual modality, as well as their combinations, influences recognition accuracy. All experiments were conducted on a standard dataset without additional noise to eliminate external variability and focus solely on the effect of input modality composition. We compared the recognition performance across various settings, including single-modality input, dual-modality combinations, and full-modality input. By observing the performance variation under these conditions, we aimed to quantify the performance gains brought by additional modality information.

**Figure 14:**
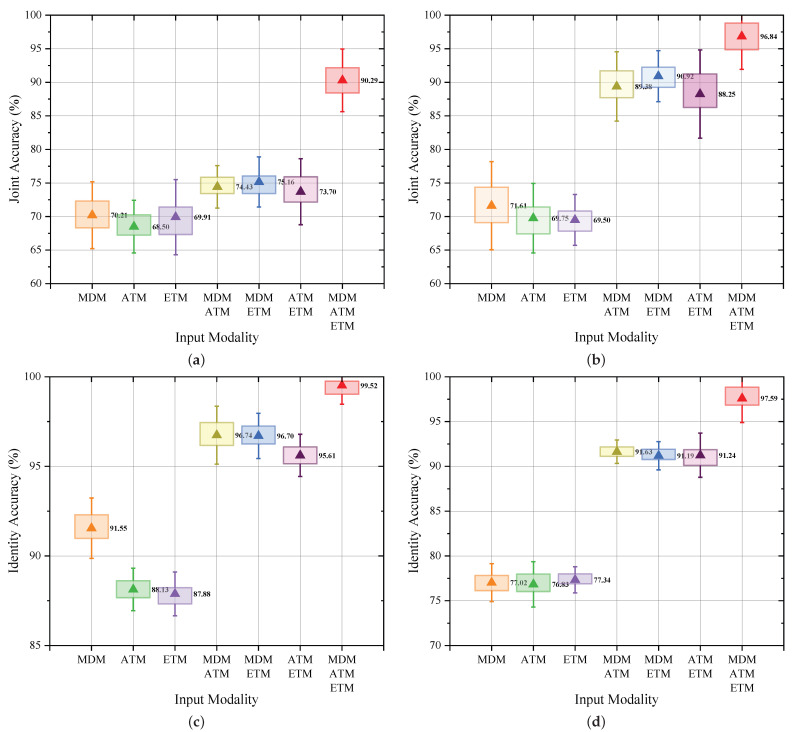
Recognition results under different input modalities. (**a**) Joint recognition accuracy of CBAM-CNN. (**b**) Joint recognition accuracy of the proposed JRF-CMAF. (**c**) Gesture recognition accuracy of the proposed JRF-CMAF. (**d**) Identity recognition accuracy of the proposed JRF-CMAF.

As depicted in Figure 14a,b, the experimental results demonstrate a clear upward trend in recognition accuracy for both the baseline CBAM-CNN and the proposed JRF-CMAF as the number of input radar modalities increases, highlighting the significance of multimodal radar information in enhancing the performance of joint gesture and identity recognition tasks. Notably, under single-modality input conditions, the recognition performance of JRF-CMAF remains largely comparable to that of the baseline method. However, with the inclusion of an additional modality, JRF-CMAF achieves a markedly higher recognition accuracy, thereby validating the capability of the proposed ARBs to effectively extract deep, task-relevant features that exploit the interdependencies among radar modalities.

Furthermore, to provide a more comprehensive understanding of how each radar modality contributes to the two subtasks individually, we additionally evaluated the gesture-recognition and identity-recognition accuracies under various single-modality and dual-modality input settings. The results, summarized in Figure 14c,d, reveal that the influence of modality composition differs substantially between the two tasks. For gesture recognition, the performance remains consistently high across different modality inputs, with the MDM modality alone already offering strong discriminative capability due to its rich temporal–dynamic micro-Doppler characteristics. In contrast, identity recognition exhibits a much stronger dependence on multimodal input. None of the single-modality configurations is able to fully capture the subtle individual-specific motion variations embedded in gesture execution, leading to noticeably lower identity-recognition accuracy. Only when at least two modalities are jointly provided does the accuracy exceed 90%, highlighting the essential role of complementary spatial–temporal information in modeling user-specific behavioral traits.

These findings reinforce the necessity of multimodal radar sensing for the joint gesture–identity recognition task and further validate that the ARB-enabled JRF-CMAF can effectively exploit cross-modal correlations to learn identity-sensitive deep representations, particularly when multiple radar modalities are available.

### 6.2. Ablation Studies

To validate the effectiveness of the proposed JRF-CMAF, all ARBs described in the shared feature extraction module in Section 4 were removed, leaving only the core three-layer convolutional backbone, which was then directly connected to the task-specific recognition output modules. In the modified architecture, features from different modalities are simply concatenated after their respective feature extraction branches, without any intermediate interaction or correction. The recognition performance of this simplified network across various tasks is summarized in Table 3. As shown, the model without ARBs experiences a slight drop in performance on the gesture recognition task, but a more substantial decline in identity recognition accuracy, which ultimately results in an overall joint recognition accuracy that falls slightly below that of the baseline.

**Figure 15:**
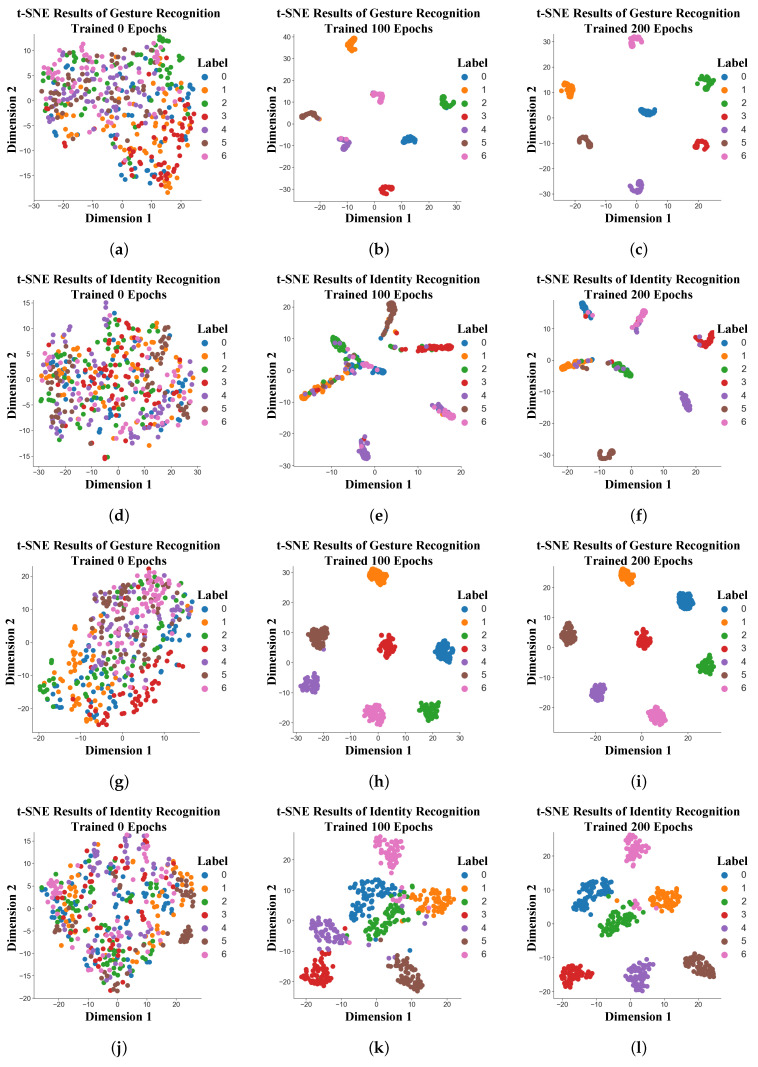
Comparative analysis of feature space formation in JRF-CMAF with and without ARBs. (**a**–**c**) Gesture recognition feature space of JRF-CMAF without ARBs. (**d**–**f**) Identity recognition feature space of JRF-CMAF without ARBs. (**g**–**i**) Gesture recognition feature space of JRF-CMAF. (**j**–**l**) Identity recognition feature space of JRF-CMAF.

Given that the primary structural difference between the two networks lies within the Shared Feature Extraction Module, this paper conducted a t-SNE clustering analysis [41] on the feature representations output by the fully connected layers after multimodal feature extraction. As illustrated in Figure 15, the two networks exhibit comparable clustering capabilities for the gesture recognition task. After 200 training epochs, both can form well-separated feature spaces for different gesture classes. However, for the identity recognition task, the proposed JRF-CMAF framework demonstrates a clear advantage. Throughout both the training process and at convergence, JRF-CMAF consistently shows superior discriminability in identity-sensitive features compared to the baseline model without ARBs. The visualization results indicate that JRF-CMAF produces more distinct inter-class boundaries and significantly lower feature overlap, reflecting improved cluster compactness and separation. These findings confirm that the ARB enhances identity feature extraction by adaptively calibrating multimodal features. Its ability to refine and align discriminative information across modalities leads to improved identity classification performance, especially in complex multi-task scenarios.

We believe this is because gesture recognition primarily relies on shallow-level features, particularly those related to micro-Doppler signatures, which can still be effectively captured through direct feature concatenation. In contrast, identity recognition depends more heavily on learning the intrinsic correlations across modalities. The absence of cross-modal correction mechanisms in this simplified architecture significantly hinders its ability to capture such relationships. This notable performance drop strongly supports the importance of the ARBs within JRF-CMAF. Their ability to effectively extract and model cross-modal dependencies plays a critical role in enhancing recognition performance, particularly for identity recognition and joint classification tasks.

### 6.3. Open-Set Evaluation on Identity Recognition

In practical gesture–identity joint recognition applications, the system may encounter additional users who were not included in the training dataset but perform the same gestures to interact with the interface. In such cases, it is desirable that the system accurately classifies the gesture type while labeling the corresponding identity as ’unknown’. This allows the HCI system to correctly interpret user intent without conflicting with personalized commands of known users.

To evaluate the adaptability of the proposed JRF-CMAF framework to such scenarios, a threshold-based rejection strategy [42] was introduced after the Softmax activation layer of the identity recognition head. In the original network, the identity category was determined by the maximum activation value among the 7 Softmax output units corresponding to the 7 enrolled subjects. With the threshold-based rejection mechanism, the predicted identity is accepted only when this maximum probability exceeds a predefined threshold; otherwise, the input is labeled as an ‘unknown’ identity. This modification enables the network to discriminate between known and unseen subjects while maintaining accurate gesture recognition.

**Figure 16:**
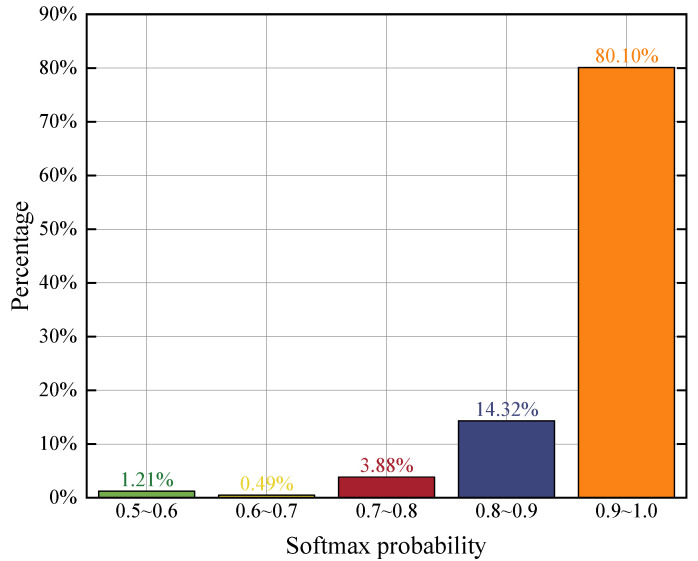
Softmax Output Distribution of the Final Classification Layer.

In the experimental design, we randomly selected gesture samples from 6 subjects in the dataset, with 80% used for training and the remaining 20% along with all data from an additional subject used for testing. As shown in Figure 16, for the 20% test samples from the executors in the training set, the probabilities from the Softmax activation function were predominantly greater than 0.5, with values mainly concentrated in the range of 0.8 to 0.9. To evaluate the identity rejection ability, we set the thresholds to 0.6, 0.7, and 0.8. The corresponding closed-set identity recognition accuracies were 96.40%, 95.91%, and 92.13%, while the identity rejection accuracies for the additional executor’s gesture data were 86.07%, 91.07%, and 91.79%, respectively. Experimental results demonstrate that, on the proposed JRF-CMAF, the application of a simple threshold-based rejection mechanism enables effective rejection of unseen gesture subjects in open-set scenarios, while incurring only a slight decrease in closed-set recognition accuracy.

### 6.4. Model Complexity Analysis

To further evaluate the computational efficiency and deployment feasibility of the proposed JRF-CMAF model, we analyzed and compared the number of parameters and the computational complexity (FLOPs) of all evaluated models, as summarized in Table 4. These two indicators are critical for assessing the trade-off between model performance and hardware resource consumption. As shown, the proposed JRF-CMAF achieves the highest recognition performance while maintaining a lightweight architecture with only 1.18M parameters and 72 MFLOPs. Compared with the baseline models, the computational complexity of JRF-CMAF is slightly higher than that of MRN but significantly lower than that of CBAM-CNN and other transformer-based and RNN-based methods. The inclusion of the ARB module slightly increases the computational cost but introduces negligible growth in parameter count, indicating that the performance improvement mainly originates from more effective feature aggregation rather than model expansion.

Furthermore, we conducted inference experiments on an embedded platform (NVIDIA Jetson Nano). The results show that JRF-CMAF requires only 330 μs to complete one forward pass for joint gesture and identity recognition, demonstrating that the model fully meets the real-time requirements of radar-based multimodal interaction systems. These findings verify that JRF-CMAF not only achieves superior recognition accuracy but also maintains excellent computational efficiency, making it highly suitable for edge and embedded radar deployment scenarios.

## 7. Conclusions

This paper presents JRF-CMAF, a unified framework for joint gesture and identity recognition using multimodal features extracted from mmWave radar echoes. The system leverages a TDM-MIMO radar to acquire gesture data, followed by a robust preprocessing pipeline, including zero-velocity filtering, gesture range detection, and valid frame selection, to isolate meaningful motion information. It then extracts multimodal features including MDMs, ETMs, and ATMs. A novel network architecture, equipped with cross-modal ARBs, is designed to enhance inter-modal feature alignment and robustness. Extensive evaluations on a self-constructed dataset demonstrate that JRF-CMAF achieves state-of-the-art performance in recognition accuracy. Overall, JRF-CMAF provides a scalable and reliable solution for radar-based gesture–identity joint recognition.

## Figures and Tables

**Table 1:** Radar experimental platform parameter setting.

Parameter	Value
Modulation bandwidth (*B*)	4 GHz
Carrier frequency ($f_{c}$)	60 GHz
Sampling frequency ($f_{s}$)	2.5 MHz
Chirp duration ($T_{c}$)	134 μs
Chirp slope (*S*)	39 MHz/μs
Number of transmitting antennas	4
Number of receiving antennas	4

**Table 2:** Illustrations of gestures and their multimodal radar feature maps.

Gesture	Gesture Name (Index)	MDM	ATM	ETM
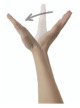	Waving left (G1)	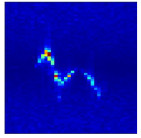	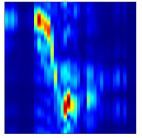	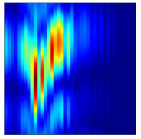
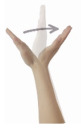	Waving right (G2)	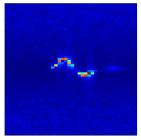	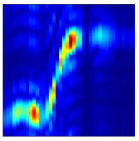	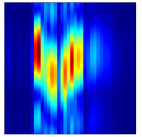
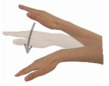	Waving down (G3)	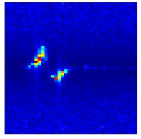	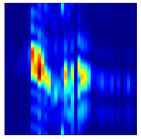	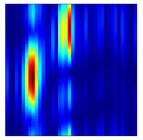
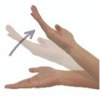	Waving up (G4)	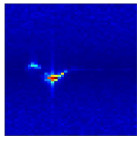	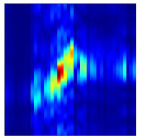	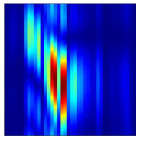
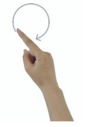	Rotating clockwise (G5)	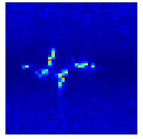	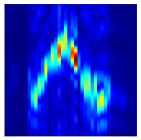	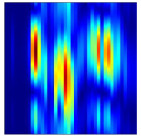
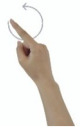	Rotating counterclockwise (G6)	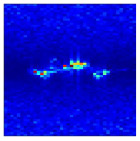	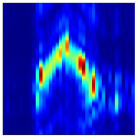	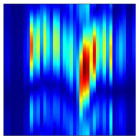
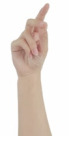	Snapping (G7)	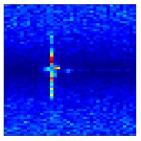	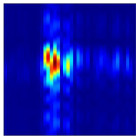	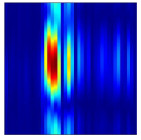

**Table 3:** Hyperparameter setting in training process.

Parameter	Value
Optimizer	Adam
Learning Rate	$1\times 10^{-5}$
Weight Decay	$1\times 10^{-4}$
Dropout	0.3
Batch Size	64
Epoch	200
Initialized Weights	Gaussian Random

**Table 4:** Comparison of recognition accuracy and computational complexity of different joint recognition task algorithms.

Models	Gesture	Identity	Joint Task	Prarms	FLOPs
MN-UIV [34]	90.85%	88.56%	85.04%	10.64M	230M
JMI-CNN [40]	98.50%	80.92%	80.52%	6.43M	180M
MRN [38]	96.62%	74.26%	73.11%	1.02M	68M
CBAM-CNN [39]	92.23%	93.30%	90.29%	14.4M	1490M
JRF-CMAF without ARBs	98.21%	89.80%	89.32%	1.16M	21M
JRF-CMAF	99.76%	97.57%	96.84%	1.18M	72M

## Data Availability

The original contributions presented in the study are included in the article, further inquiries can be directed to the corresponding author.
